# Fluoride Casein Phosphopeptide and Tri-Calcium Phosphate Treatments for Enamel Remineralization: Effects on Surface Properties and Biofilm Resistance

**DOI:** 10.3390/dj13060246

**Published:** 2025-05-30

**Authors:** Cecilia Carlota Barrera-Ortega, Sandra E. Rodil, Phaedra Silva-Bermudez, Arturo Delgado-Cardona, Argelia Almaguer-Flores, Gina Prado-Prone

**Affiliations:** 1 Research Laboratory in Nano and Dental Biomaterials, Faculty of Higher Studies Iztacala (FESI), National Autonomous University of Mexico (UNAM), Mexico City 54090, Mexico; 2 Materials Research Institute, National Autonomous University of Mexico (UNAM), Mexico City 54090, Mexico; srodil@unam.mx (S.E.R.); jadcardona95@gmail.com (A.D.-C.); 3 Unidad de Ingeniería de Tejidos, Terapia Celular y Medicina Regenerativa, Instituto Nacional de Rehabilitación Luis Guillermo Ibarra Ibarra, Calzada Mexico-Xochimilco No. 289, Col. Arenal de Guadalupe, Mexico City 14389, Mexico; pssilva@inr.gob.mx; 4 Biointerfases Laboratory, Division of Graduate Studies and Research, Faculty of Dentistry, National Autonomous University of Mexico (UNAM), Circuito Exterior s/n, Ciudad Universitaria, Mexico City 04510, Mexico; argelia.almaguer@mac.com (A.A.-F.); gpradoprone@comunidad.unam.mx (G.P.-P.)

**Keywords:** CPP-ACP-F, β-TCP-F, enamel remineralization, surface hardness, roughness, wettability, biofilm

## Abstract

**Objectives:** This study aimed to compare in vitro the protective effect of two enamel remineralizing agents, a varnish containing β-tricalcium phosphate with sodium fluoride (β-TCP-F) and a paste containing casein phosphopeptide-amorphous calcium phosphate with sodium fluoride (CPP-ACP-F), on artificially demineralized human enamel. **Methods:** A total of 120 human third molar enamel specimens were randomly assigned to four groups (n = 30 each): Group I (healthy enamel, control), Group II (initially demineralized, lesioned enamel), Group III (demineralized enamel and treated with β-TCP-F), and Group IV (demineralized enamel and treated with CPP-ACP-F). Groups II–IV underwent, for 15 days, a daily pH cycling regimen consisting of 21 h of demineralization under pH 4.4, followed by 3 h of remineralization under pH 7. Groups III and IV were treated with either β-TCP-F or CPP-ACP-F, prior to each 24 h demineralization–remineralization cycle. Fluoride ion release was measured after each pH cycle. Surface hardness, roughness, wettability, and *Streptococcus mutans* biofilm formation were assessed on days 5, 10, and 15 after a daily pH cycle. **Results:** CPP-ACP-F treatment showed a larger improvement in surface hardness (515.2 ± 10.7) compared to β-TCP-F (473.6 ± 12.8). Surface roughness decreased for both treatments compared to initially lesioned enamel; however, the decrease in roughness in the β-TCP-F group only reached a value of 1.193 μm after 15 days of treatment, a significantly larger value in comparison to healthy enamel. On the other hand, the decrease in roughness in the CPP-ACP-F treatment group reached a value of 0.76 μm, similar to that of healthy enamel. Contact angle measurements indicated that wettability increased in both treatment groups (β-TCP-F: 71.01°, CPP-ACP-F: 65.24°) compared to initially lesioned samples in Group II, reaching WCA values similar to or smaller than those of healthy enamel surfaces. **Conclusions**: Both treatments, β-TCP-F and CPP-ACP-F, demonstrated protective effects against enamel demineralization, with CPP-ACP-F showing superior enhancement of surface hardness and smoother enamel texture under in vitro pH cycling conditions. β-TCP-F varnish and CPP-ACP-F paste treatments counteracted surface modifications produced on human healthy enamel by in vitro demineralization.

## 1. Introduction

Dental enamel is the outermost layer of tooth crowns, the hardest and most mineralized tissue in the human body. It is a composite material of approximately 95% mineral phase, predominantly hydroxyapatite, Ca_10_(PO_4_)_6_(OH)_2_; 1% organic phase, mainly formed by proteins and lipids; and 4% water. Its composition and structure enable dental enamel to withstand a wide range of physical and chemical demands in the oral environment, including significant compressive forces and pH fluctuations [1]. The mineral phase of dental enamel is susceptible to dissolution in acid solutions (pH < 5.5), leading to a loss of calcium, phosphate, and other ions; a phenomenon known as demineralization [2]. Saliva has the natural ability to remineralize dental enamel by supplying calcium and phosphate ions; however, this natural remineralization process is not enough when the pH in the oral cavity tends to remain acidic for long periods of time [3]. Consequently, persistent acidic conditions in the oral cavity, mainly caused by dietary habits and oral bacteria, result in permanent demineralization of the dental enamel, causing alterations in its surface properties, facilitating bacterial adhesion on the teeth surface, and finally contributing to an increase in oral conditions such as caries or dental erosion [4].

According to the World Health Organization, dental caries (also referred to as dental cavities or tooth decay) is the most prevalent noncommunicable disease, affecting 60–90% of school-age children and most adults. It is considered a major public health concern that consumes 5–10% of the healthcare budget in industrialized countries [5]. Caries lesions usually begin with permanent demineralization of dental enamel; thus, various strategies for remineralization of dental enamel have been recently developed to enhance or preserve oral health. Treatments frequently promote early enamel remineralization by driving precipitation and solidification of minerals onto the enamel surface from ion clusters in saturated solutions [6]. Among the different remineralization strategies, the use of fluorine-based compounds is one of the most common strategies [7]. Topical application of fluoride products, such as sodium fluoride (NaF), has been demonstrated to promote remineralization of non-cavitated enamel lesions; however, fluoride ion-promoted remineralization is limited by the availability of calcium and phosphate ions in the microenvironment [8]. Thus, enough calcium and phosphate ions must be present in the oral microenvironment for fluorine-based product treatments to function correctly and promote remineralization.

Two of the most widely used fluorine-based remineralizing agents in pediatric dentistry are (a) varnishes containing beta-tricalcium phosphate (β-TCP) along with sodium fluoride (NaF; 22,600 ppm) and (b) pastes containing casein phosphopeptide (CPP) and amorphous calcium phosphate (ACP) along with NaF, where casein phosphopeptide, a milk-derived protein, stabilizes calcium phosphate in solution and facilitates its binding to enamel. For both fluorine-based products, fluoride enhances remineralization in the form of NaF, contributing to caries prevention [9]. A dentist applies β-TCP-F varnishes in dental practice, while CPP-ACP-F pastes can be applied daily at home. The general population can freely choose between these two alternatives, so it is important to further assess the effectiveness of these two widely used remineralizing agents to better establish and understand their benefits and limitations [10].

In the case of β-TCP-F-containing varnishes, they function as calcium and phosphate ion reservoirs, regulating the transfer of these ions into the teeth surface and synergistically enhancing remineralization alongside fluoride ions. Furthermore, the interaction among these ions in the oral environment can favor the fluorapatite receptor, which enhances enamel’s resistance to demineralization. Moreover, NaF prevents undesirable reactions between calcium and phosphate ions during storage. Upon contact with saliva, the protective varnish barrier dissolves gradually, releasing its ions over time for effective tooth remineralization [11,12]. The United States Food and Drug Administration (FDA) approves the use of cavity varnishes to treat sensitive teeth, especially on exposed dentin tissue and the tooth root. Varnishes are also approved for application into cavities right before the insertion of restorative materials to prevent their penetration into the dentin tissue. Fluoride varnishes are recognized as an effective treatment not only for hypersensitivity but also in cases of patients with a history of caries. One of the most commonly used cavity varnishes in pediatric dentistry is the Vanish^®^ (St. Paul, MN, USA) β-TCP-F system, which is mainly used for three reasons: (1) its properties have been designed to provide sustained release of ions, not only to the teeth in close contact with the varnish, but also within the whole oral cavity; (2) it is supplemented with an innovative form of β-TCP-F; and (3) it is easy and quick to apply in the dental practice.

Proteins like caseins regulate the solubility of calcium and phosphate ions in biological systems [13], and high concentrations of these ions with no regulation can lead to undesirable calcifications. Thus, fluorine-based pastes containing CPP and ACP can favor controlled remineralization, avoiding undesirable calcification side effects. Casein is the major protein present in milk. It can bind to calcium and phosphate ions, forming stable complexes that can be released, by partial enzymatic digestion, in the form of CPP sequences [14]. The CPP-ACP-F systems have been demonstrated to prevent tooth enamel demineralization and dental erosion [15,16]. When the pH in the oral cavity decreases, the CPP-ACP-F system releases Ca^2+^ and PO_4_^3−^, creating a supersaturated state of these ions around teeth [17], which is then stabilized by CPP at 4.5–7 pH values, forming minerals consistent with fluorapatite in the presence of fluoride ions [18]. The CPP-ACP-F system has been shown to delay biofilm formation and favor the crystallization of calcium phosphates [17,19,20,21,22], inhibiting the demineralization of tooth enamel and consequently preventing tooth decay [23].

Currently, there are different commercial gels, varnishes, and pastes aimed at promoting tooth enamel remineralization. Although many have demonstrated good efficacy, further research is still needed to support their use and evaluate their effects on the various complementary properties of tooth enamel. In the present study, we focused on comparing the efficacy of a varnish containing β-TCP-F and a paste containing CPP-ACP-F, as these are two representative products of the different enamel remineralization systems available on the market [24].

This work aimed to comparatively evaluate the in vitro effect on the surface of demineralized human dental enamel of two of the most used fluorinated compounds in pediatric dentistry, that is, a varnish containing β-tricalcium phosphate with sodium fluoride (β-TCP-F) and a paste containing casein phosphopeptide-amorphous calcium phosphate with sodium fluoride (CPP-ACP-F). Different methods exist to study the in vitro demineralization and remineralization of dental enamel at different intervals, such as the cyclical pH model, which simulates pH daily cycles in the oral environment [25,26]. Thus, after initial demineralization, demineralized enamel tooth surfaces were daily treated with either β-TCP-F or CPP-ACP-F and subjected to 21–3 h demineralization–remineralization pH cycles for 5, 10, and 15 days; finally the hardness, roughness, and wettability of the enamel surfaces were studied at each time interval (5, 10, or 15 days) and compared with those of healthy enamel surfaces. Demineralized enamel surfaces that received no treatment, neither β-TCP-F nor CPP-ACP-F, were subjected to a daily pH demineralization–remineralization cycle and used as negative comparative controls. Furthermore, the presence of fluoride ions was daily measured in the aqueous environment where experimental treated surfaces were subjected to demineralizing–remineralizing pH cycles. Finally, the susceptibility of the experimental enamel surfaces to biofilm formation was studied using the cariogenic bacteria *Streptococcus mutans*.

This study shows the varying efficacy of two widely used fluorine-based products in mitigating the effects of demineralization on human dental enamel surfaces, including susceptibility to biofilm formation. Thus, it represents a valuable tool for dentists to make evidence-based decisions.

## 2. Materials and Methods

### 2.1. Obtention of Human Dental Enamel Specimens

The enamel samples were obtained from lower retained third molars from 17- to 22-year-old patients who needed orthodontic or preventive treatment and to whom third molars extraction surgery was recommended. As retained third molars were not exposed to the oral cavity before extraction surgery, the risk of contamination was significantly reduced. The third molars studied were obtained from donors undergoing dental extraction procedures who had informed consent, through donations from maxillofacial surgeons from different private clinics. All molars were obtained over three months. This study was approved by the Ethics Committee of the Faculty of Higher Studies (FES) Iztacala (CE/FESI/032022/1499, approved on 1 January 2022) and followed the Declaration of Helsinki.

### 2.2. Preparation of Experimental Groups

This study used 60 human third molars with intact crowns and no structural defects (i.e., molars presented healthy tooth enamel). Third molars obtained were cut in half longitudinally in a mesiodistal direction with a diamond disk (Brasseler™ diamond 910 < 20,000 rpm, Savannah, GA, USA), under constant irrigation to obtain 120 working surfaces; periodontal debris was removed before teeth were sectioned. The working specimens were filled with pink wax in the internal area of the tooth (pulp chamber and root canals) to obtain a flat back-surface and stored in deionized, distilled water until experimental tests were performed. Following Ten Cate [19], non-fluorinated prophylactic paste (Viarden Lab, Mission, TX, USA) prophylaxis was undertaken. Working surfaces were finished by covering the root and crown with acid-resistant varnish (Revlon™, different colors, New York, NY, USA), leaving a 3 × 6 mm^2^ area exposed in each sample at the center of the lingual or buccal surface of the anatomic crown. Thirty working samples were left as obtained and used as healthy enamel controls (Group I: HE), while the remaining 90 surfaces were lesioned with demineralizing solution for 96 h according to the pH cycling model by Ten Cate and Duijsters [21], one of the best models for reproducing in vivo oral conditions and the most widely used model to study the loss and gain of minerals on artificially lesioned enamel. Briefly, the 90 enamel working surfaces were immersed in a demineralizing solution (2.2 mM CaCl_2_, 2.2 mM NaH_2_PO_4_, and 0.05 M CH_3_COOH) for 96 h at 37 °C, while the pH was adjusted to 4.4 with 1M KOH solution. Before beginning the experimental phase, each sample was immersed in a solution of distilled, deionized, and demineralized water in a temperature-controlled chamber at 36 °C to prevent changes in the enamel’s mineral content.

Then, 30 working surfaces were daily treated with the paste containing casein phosphopeptide-amorphous calcium phosphate with NaF (Mi Paste Plus™ GC IBÉRICA Dental Products, S.L. Edificio Codesa 2 Playa de las Americas, 2, 1°, Of. 4, 28290 Las Rozas, Madrid, España); the other 30 working surfaces were treated with the varnish containing β-tricalcium phosphate with NaF (Clinpro™ White Varnish. St. Paul, MN, USA), following the manufacturer’s instructions; and the last 30 working surfaces were left as demineralized and used as untreated, lesioned, negative control enamel samples.

**Figure 1:**
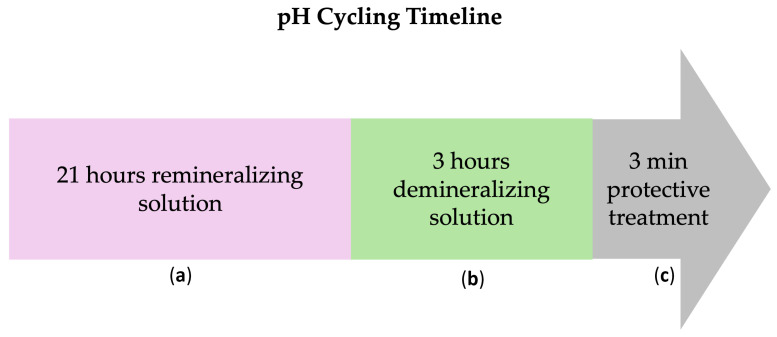
Daily pH–treatment cycles to which working samples surfaces were exposed for 5, 10, and 15 days: (**a**) 21 h in remineralizing solution (1.5 mM CaCl_2_, 0.9 mM NaH_2_PO_4_, and 0.15 mM KCl with pH adjusted to 7.0 with 1 M KOH buffer solution); (**b**) 3 h in demineralizing solution (2.2 mM Ca, 2.2 mM P, and 5.0 mM CH_3_COOH with pH of 4.4); and (**c**) for treated working surfaces, a 3 min treatment application of either β-TCP-F varnish or CPP-ACP-F paste.

Subsequently, all working surfaces (either untreated or treated with β-TCP-F or CPP-ACP) were subjected to daily in vitro pH cycles, alternating demineralizing (pH = 4.4, for 21 h) and remineralizing (pH = 7, for 3 h) solutions, to mimic the in vivo oral conditions (Figure 1). These cycles were performed daily for 5, 10, and 15 days using fresh pH solutions. At each measurement time (5, 10, and 15 days), 10 surfaces of each group were randomly chosen and immersed in deionized water at 37 °C, and their surface properties and susceptibility to biofilm formation were studied. Five days is equivalent to 1.2 months of remineralization treatment, 10 days to 2.5 months of remineralization treatment, and 15 days to 3.8 months of remineralization treatment. Considering this, a once-a-week application of the remineralization treatment can be used in cases of severe demineralization [27,28].

**Figure 2:**
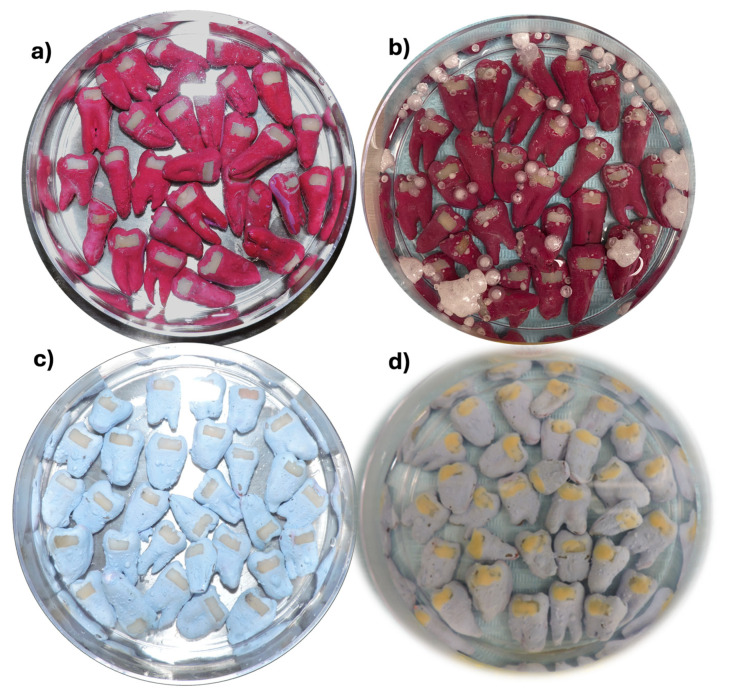
Different treatment working surfaces used in the present study, (**a**) before and (**b**) after treatment with β-TCP-F, and (**c**) before and (**d**) after treatment with CPP-ACP-F.

Working surfaces were categorized into three groups (Figure 2) according to their treatment: Group II: initially lesioned and no treatment (IL, [n = 30]); Group III: initially lesioned and treated with β-tricalcium phosphate with NaF (β-TCP-F, [n = 30]); and Group IV: initially lesioned and treated with casein phosphopeptide-amorphous calcium phosphate with NaF (CPP-ACP-F, [n = 30]).

### 2.3. Fluoride Ion Content in the Demineralizing–Remineralizing pH Cycling Solutions

The amount of fluoride ions (ppm) in the demineralizing–remineralizing pH solutions was measured daily during the cyclical pH treatment using a Fluoride-Ion Selective Electrode (F-ISE) (Orion Star A-214^®^, Orion Research, Austin, TX, USA) and a pH/Ion 450/M^®^ fluorimeter (Techno Scientific, Waltham, MA, USA). For electrode calibration, calibration curves from 0.250 to 2 μg/mL were constructed using Tissab II^®^ solutions (Orion, Techno Scientific, Waltham, MA, USA), which hold the ions stable, raise the pH value, and release fluoride ions bound to metal ions. Measurements were performed at room temperature, in Tissab II^®^ solution (Orion, Techno Scientific, Waltham, MA, USA), which keeps the ions stable, raises the pH value, and releases fluoride ions that are bound to the metal ions (iron, calcium, aluminum, etc.), preventing their interference in fluoride measurements. Fluoride concentration in the samples was determined by interpolating the calibration curves with a range of 2 to 0.25 μg/mL. Units of values reported correspond to millivolts.

To perform the measurements, the solutions were independently transferred to glass containers (Sigma-Merck, Darmstadt, Germany) using a dropper and mixed at room temperature (≈25 °C) with a magnetic stirrer. Reading was registered when the solution reached a stabilized state. The electrodes were rinsed with deionized water and dried with paper towels (Sanitas™, Madrid, Spain) between each measurement. New calibration curves were prepared daily to ensure the reproducibility of the results within ±2%.

### 2.4. Vickers Hardness

The Vickers hardness of 10 working surfaces from each group after 0, 5, 10, and 15 days of pH cycling treatment was determined using a durometer (Nano-Microindentador, NANOVEA Inc. Headquarters. Irvine, CA, USA; software Nanovea Micro Indentation Tester v1.8.3). Using a 20× objective lens, the indenter was placed perpendicularly to the working surface of each sample, and the assays were performed with the following conditions: approach speed: 50 μm/min; contact load: 50 mN; load: 10 N; loading rate: 5 N/min; and unloading rate: 5 N/min, with 10 notches per sample. The Vickers hardness number (VHN) was calculated using the Nanovea Micro Indentation Tester™ (NANOVEA Inc. Headquarters. Irvine, CA, USA) software [29].

### 2.5. Surface Roughness

The roughness of the surfaces was measured using a ZYGO 3D-Nexview (Zygo, Middlefield, CT, USA) non-contact optical profilometer. Average roughness, Ra, which is defined as the arithmetic mean of the absolute values of the surface profile deviations from the mean line, was used to report the surface roughness. It measures the average distance between the surface’s peaks and valleys over a given evaluation length and is the most widely reported roughness value in materials science. For this, six roughness measurements (Ra) were randomly recorded on the 3 × 6 mm^2^ working surface of each sample, and the average of the profilometry data was obtained. Surface roughness measurements were recorded at 0, 5, 10, and 15 days of pH cycling treatment, and significant differences were calculated by comparing experimental treated groups’ measurements vs. Group I (Healthy Enamel; HE) and Group II (Initial Lesion, not treated; IL).

### 2.6. Wettability

The wettability of the surfaces was analyzed by measuring their water contact angle, using a goniometer (Dataphysics OCA-15EC. DataPhysics Instruments, Filderstadt, Germany). Two measurements were performed for each sample, placing a sessile drop of deionized water (4 μL) on the 3 × 6 mm^2^ working surface. The contact angle was determined using the SCA20_U software (V.5.0.38 build 5038), one second after the water droplet on the surface was stable, with 20 measurements performed for each sample in all groups, taking 10 contact angles on the left side and 10 on the right side as references.

### 2.7. Scanning Electron Microscopy

The samples were morphologically characterized by field emission scanning electron microscopy (FE-SEM, JEOL JSM-7600F, JEOL Ltd., Tokyo, Japan). Before SEM observation, samples were coated with a thin conductive carbon layer using a vacuum coater to avoid artifacts and ensure adequate electrical conductivity. Samples were analyzed under high vacuum conditions, at an acceleration voltage of 20 kV and a magnification of 5000×, using the secondary electron detector to obtain high-resolution images that allowed a representative and accurate surface topography evaluation.

### 2.8. Susceptibility to Streptococcus mutans Biofilm Formation

Biofilm formation of the cariogenic bacteria *Streptococcus mutans* (*S. mutans*; 25175™, ATCC^®^, Manassas, VA, USA) on dental enamel treated with β-TCP-F or CPP-ACP-F for 5, 10, and 15 days was evaluated by the MTT assay (Sigma-Aldrich. Merck KGaA, Darmstadt, Germany). For this, a pure culture of *S. mutans* was collected from a Petri dish prepared with HK agar (Trypticase Soy Agar, Brain Heart Infusion Agar, and Yeast, acquired from BBL^®^) enriched with menadione 1% *v*/*v* (Sigma-Aldrich, Merck KGaA, Darmstadt, Germany), hemin 1% *v*/*v* (Sigma-Aldrich, Merck KGaA, Darmstadt, Germany), and 5% of defibrinated sheep blood (Microlab Laboratory S.A. de C.V., Tlalnepantla, Mexico) and resuspended in Mycoplasma Broth Base (BBL^®^, Sigma. Saint Louis, MO, USA) enriched with menadione 1% *v*/*v* and hemin 1% *v*/*v*. A bacterial suspension of 1 × 10^9^ cells/mL was obtained by adjusting the suspension to an optical density (OD) of 1 at λ = 600 nm, using a spectrophotometer (BioPhotometer D30. Fisher Scientific. 28108, Alcobendas, Madrid, Spain). Then, steam-sterilized surface samples (three from each group) were placed in 24-well culture plates, and 2 mL of a bacterial suspension at 1 × 10^7^ cells/mL was inoculated in each well. Healthy dental enamel (Group I: HE) and demineralized dental enamel (Group II: IL) were used as positive and negative control groups, respectively. Inoculated samples were incubated for 24 h at 37 °C and 80 rpm in an orbital shaker (IKA^®^ KS 130 basic, Staufen, Germany) under anaerobic conditions. After the incubation period, the samples were rinsed twice with steam-sterilized double-distilled water (dd-H_2_O) to detach loosely attached bacteria, transferred to a new culture well plate, and incubated with a 1:10 solution of MTT–culture broth for 3 h at 37 °C and 80 rpm, under dark and anaerobic conditions. Then, the formazan crystals metabolized by the viable bacterial attached on the samples were solubilized in a 1:1 solution of 2-propanol 99% (ISO; Sigma-Aldrich, Merck KGaA, Darmstadt, Germany) and dimethyl sulfoxide (DMSO; Sigma-Aldrich, Merck KGaA, Darmstadt, Germany), and the OD was read at λ = 570 nm (Filter-MaxF5 multi-mode microplate reader; Molecular Devices, San Jose, CA, USA) to determine the amount of viable bacterial attached to the surfaces and, consequently, their susceptibility for biofilm formation.

### 2.9. Statistical Analysis

The Shapiro–Wilk test was performed to analyze the data distribution of the F-ISE variables (ppm of fluoride ions), hardness, and roughness. Non-parametric Kruskal–Wallis tests were performed for the F-ISE and hardness data, and multiple comparisons were made using Wilcoxon’s Rank and Dunn’s Test, respectively. Intergroup comparison for roughness measurements was performed with one-way analysis of variance (ANOVA) followed by pairwise comparison using Tukey’s honestly significant difference (HSD) post hoc test. Bacterial experiments were performed in triplicate, and the results were presented as the mean values (ME) ± the standard error of the mean (SEM); data were analyzed using the one-way analysis of variance (ANOVA) followed by Tukey’s multiple comparison test. In all tests, *p* < 0.05 was considered statistically significant. All statistical analyses and graphs were performed with the GraphPad Software Prism 8.0^®^ (225 Franklin Street. Fl. 26, Boston, MA, USA).

## 3. Results

### 3.1. Fluoride Ion Content in Demineralizing and Remineralizing Solutions

**Figure 3:**
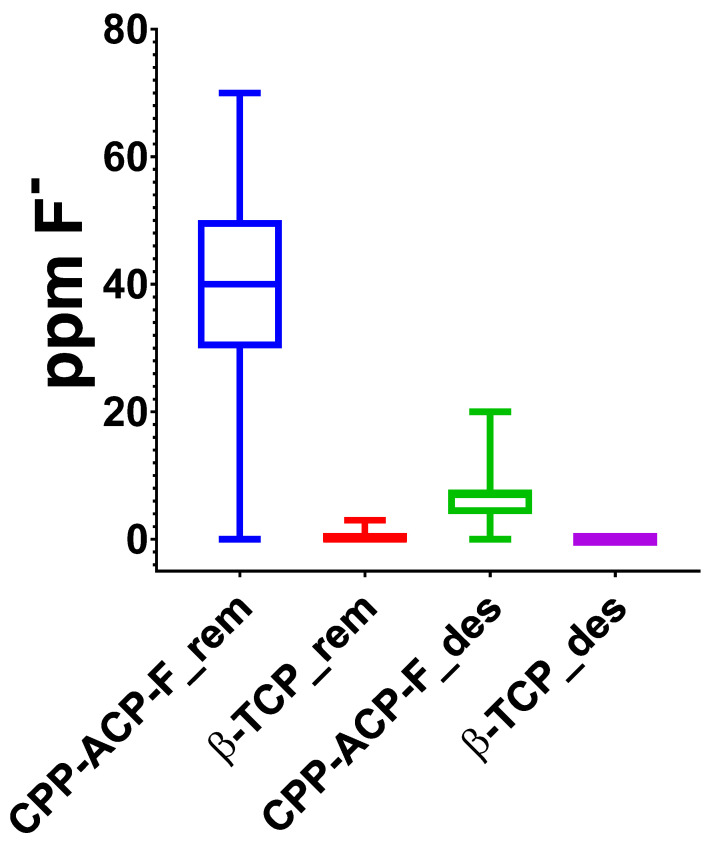
Box and whisker plot of the cumulative fluoride ions released from varnish and paste treatment throughout the 15-day cycling pH treatment.

The cumulative concentration of fluoride ions (ppm) in the mineralizing and demineralizing solutions used over the 15 days of cycling pH to which the different human enamel experimental surfaces were subjected are shown in Figure 3. It can be observed that remineralizing and demineralizing solutions used for the samples treated with β-TCP-F contained a higher amount of fluoride ions compared to the solutions used for the CPP-ACP-F-treated surfaces; the remineralizing solutions (pH = 7, 21 h of surface immersion) contained a higher fluoride ions concentration compared to the demineralizing solutions (pH = 4.4, 3 h of surfaces immersion).

### 3.2. Vickers Hardness

**Figure 4:**
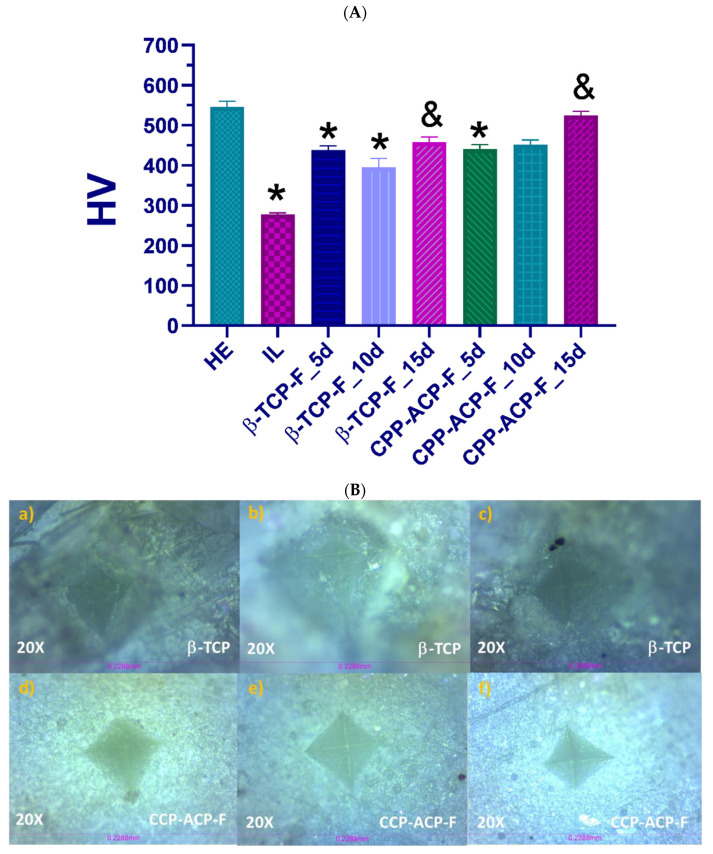
(**A**) Vickers hardness measurements on the enamel surfaces of each group. *: *p* < 0.05, any surface vs. healthy dental enamel (Group I: HE; positive control). &: *p* < 0.05, any surface vs. demineralized dental enamel (Group II: IL; negative control). (**B**) Representative images of the surfaces after the hardness test; surfaces treated with β-TCP-F for (**a**) 5 days, (**b**) 10 days, and (**c**) 15 days; and surfaces treated with CPP-ACP-F for (**d**) 5 days, (**e**) 10 days, and (**f**) 15 days.

Table 1 presents the mean values of the Vickers hardness number (HV ± SD) of the healthy enamel, Group I, and experimental groups, either untreated (Group II) and treated with β−TCP-F varnish (Group III) or CPP-ACP-F paste (Group IV) after 5, 10, and 15 days of demineralizing–remineralizing pH cycling. The mean HV value ± SD with respective significant differences and the representative images of indentations produced on the surfaces of the experimental groups are shown in Figure 4.

The hardness of the healthy enamel group (Group I: HE = 533.8 ± 14.8 HV) was significantly reduced after the initial demineralizing lesion (Group II: IL = 281.0 ± 04.1 HV). Compared with initially demineralized enamel (Group II), all experimental treated groups showed higher hardness values from 406.3 to 515.2 HV after treatments with β−TCP-F and CPP-ACP-F over pH cycling periods. However, the increment of enamel hardness for the treated surfaces was significant only after 15 days of being treated with both compositions and pH cycling, reaching 473.6 ± 12.8 HV for enamel treated with β−TCP-F and 515.2 ± 10.7 HV for enamel treated with CPP-ACP-F.

### 3.3. Roughness Surface

**Figure 5:**
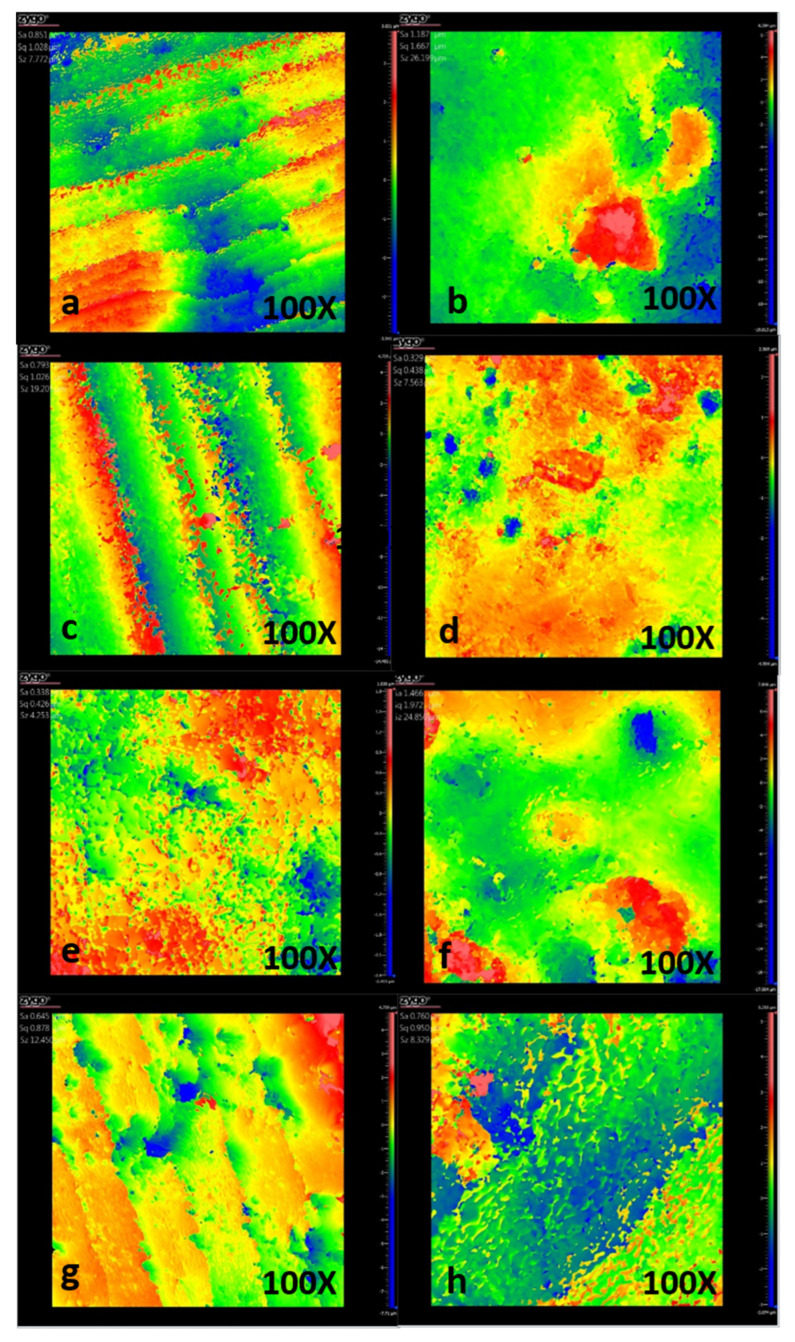
Representative profilometry images of each experimental group: (**a**) healthy enamel (HE); (**b**) initial lesion (IL); (**c**) CPP-ACP-F, 5-day treatment; (**d**) CPP-ACP-F, 10-day treatment; (**e**) CPP-ACP-F, 15-day treatment; (**f**) β-TCP-F, 5-day treatment; (**g**) β-TCP-F, 10-day treatment; and (**h**) β-TCP-F, 15-day treatment.

The mean of roughness measurements (Ra ± SD) of the comparative control Groups I and II, and experimental Groups III and IV, are shown in Table 2. Representative profilometry images of the surface roughness of the samples are shown in Figure 5. Initially demineralized enamel (Group II: IL) exhibited a greater surface roughness, with a Ra value of 1.33 ± 0.14 μm, compared to healthy enamel (Group II: HE) with a Ra value of 0.88 ± 0.08 μm. After 5 days of treatment with β-TCP-F, the roughness of the injured enamel remained similar (Ra = 1.358 ± 0.25 μm) to the initially lesioned enamel; however, as treatment days increased, the roughness gradually decreased, reaching Ra values of 1.29 ± 0.13 μm at 10 days of β-TCP-F treatment and 1.193 ± 0.14 μm after 15 days of β-TCP-F treatment. Conversely, treatment with CPP-ACP-F resulted in a considerable reduction in the roughness of lesioned enamel from the first 5 days of treatment (Ra = 0.72 ± 0.05 μm), which was maintained through the 15 days of treatment (Ra = 0.76 ± 0.05 μm), reaching similar roughness values to those of healthy enamel.

### 3.4. Wettability

**Figure 6:**
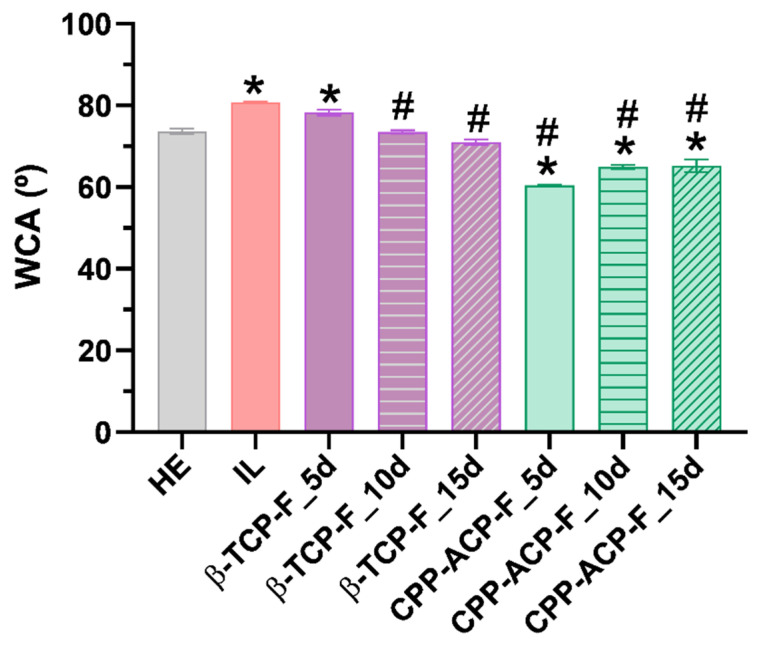
Water contact angles (°) results (mean ± SD) of the human enamel specimens of different groups, * and # indicate significant difference, *p* < 0.05.

Water contact angle measurements for the enamel surfaces are presented as mean ± SD in Table 3 and are graphically depicted in Figure 6. All enamel surfaces, comparative controls, and experimental treatments exhibited a hydrophilic character (i.e., wettability to water) as indicated by water contact angle (WCA) measurements smaller than 90°. However, differences in wettability were observed among the groups. The healthy enamel surfaces (Group II: HE) showed greater hidrophilicity with a WCA = 73.65°, compared to the lesioned enamel surfaces (Group II: IL) with a WCA of 80.82°. Following treatment with both fluoride compounds, the wettability of the lesioned enamel surfaces increased, reaching WCA values comparable to those of healthy enamel surfaces. After treatment with β-TCP-F, the wettability of the lesioned enamel surfaces showed a gradual reduction, reaching a WCA value of 71.01° after 15 days of treatment. In contrast, the wettability of the lesioned enamel surfaces treated with CPP-ACP-F initially decreased at the first 5 days of treatment (WCA = 60.42°) and gradually increased again over the following days of treatment, reaching a WCA of 65.24° after 15 days of treatment.

### 3.5. Scanning Electron Microscope

On the surface of healthy enamel, particles measuring between 1 and 3 μm were observed (appearing brighter). In contrast, discontinuities of larger sizes (dark areas on the surface) and small cracks were observed on lesioned enamel. The lesioned enamel treated with the CPP-ACP-F paste for 15 days exhibited the presence of pores measuring approximately 5 μm. These observations suggest the potential unblocking of certain dentinal tubules, which were found to be coated with chains of small particles measuring approximately 0.5 μm and have been identified as remnants of the paste that had adhered to the surface. The heterogeneity of the surface could have contributed to the observed increase in bacterial colonization, as it would have provided a greater surface area available for adhesion.

**Figure 7:**
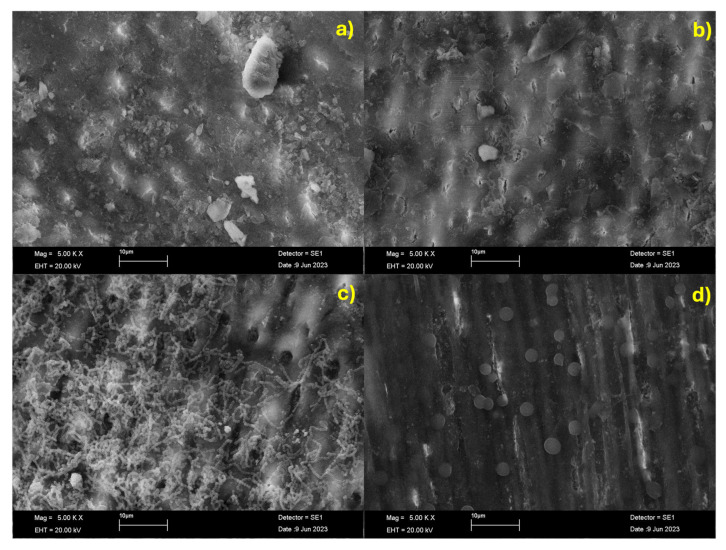
Representative SEM micrographs of (**a**) healthy enamel, (**b**) initial lesion (demineralized Enamel), (**c**) demineralized enamel treated with CPP-ACP-F for 15 days, and (**d**) demineralized enamel treated with β-TCP-F for 15 days.

The lesioned enamel treated with β-TCP-F for 15 days shows a smoother, poreless surface with a striated appearance. This may be due to the brush stroke pattern used to coat the surface with the treatment. Furthermore, small spheres are observed that are probably material residue. This poreless morphology and reduced roughness may have influenced the increase in the surface’s hardness and its ability to prevent bacterial growth over time (Figure 7).

### 3.6. Biofilm Formation

**Figure 8:**
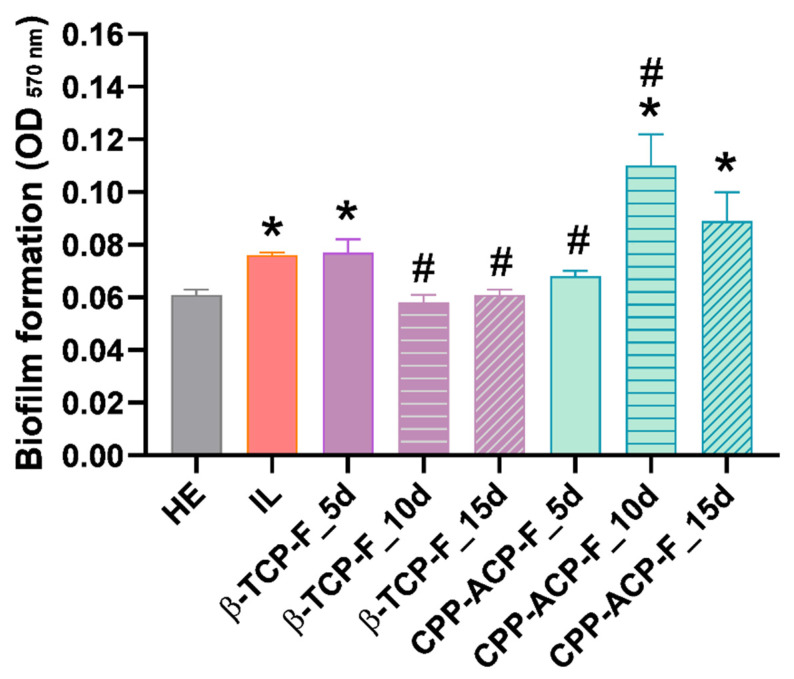
Biofilm formation of *S. mutans* at 24 h of bacterial culture on dental enamel surfaces after treatment with β-TCP-F or CPP-ACP-F for 5, 10, and 15 days, as measured by the MTT assay. * *p* < 0.05; any surface vs. Group I: HE (healthy enamel). # *p* < 0.05 any surface vs. Group II: IL (initial lesion; demineralized enamel).

Figure 8 shows the absorbance (570 nm) of formazan solutions produced by viable cariogenic bacteria *S. mutans* adhered to control and experimental dental enamel surfaces, that is, healthy, lesioned, and daily treated with β-TCP-F or CPP-ACP-F for 5, 10, and 15 days. Biofilm formation of *S. mutans* on lesioned enamel surfaces (IL) was significantly higher than that formed on healthy enamel surfaces (HE).

Lesioned enamel treated with β-TCP-F for 5 days showed no change in susceptibility to biofilm formation compared to lesioned enamel (IL), which was considered a non-protective effect. However, extending the β-TCP-F treatment to 10 and 15 days resulted in a protective effect against biofilm formation, comparable to that observed on healthy enamel surfaces (HE).

Regarding the lesioned enamel surfaces treated with CPP-ACP-F, a protective anti-biofilm effect was comparable to healthy enamel at 5 days of treatment. Nevertheless, this protective effect was not sustained after 10 and 15 days of treatment (Table 4).

## 4. Discussion

In the present study, we focused on evaluating the remineralizing effect of two fluoride-based treatments. In vitro models are essential for generating new knowledge that provides theoretical support for making initial predictions about the success of a new material, vehicle, or treatment. In vitro studies offer bioequivalence and predictions about the implementation of any product in clinical practice [30,31].

Significant advancements in remineralization therapies have focused on prolonging periods of supersaturation by delivering bioavailable calcium, phosphate, and fluoride ions to demineralized lesions, creating stable systems for ion delivery. To evaluate the efficacy of these strategies in vitro, researchers frequently employ methods like the cyclical pH model [23,25]. This model effectively simulates the fluctuating pH conditions of the oral cavity. It is based on alternating demineralizing (pH 4.4 for 21 h) and remineralizing (pH 7.0 for 3 h) solutions [18,21]. The cyclical pH method is a widely accepted approach for reproducing the demineralization and remineralization dynamics observed in vivo [26]. In this study, we utilized this well-established cyclical pH model, chosen for its precise control and ability to minimize variability while requiring a smaller sample size. This model allowed us to investigate the in vitro effects of two prominent fluorine-based remineralizing agents in pediatric dentistry: β-TCP-F and CPP-ACP-F. These agents were delivered through two distinct vehicles: a paste containing CPP-ACP and 900 ppm sodium fluoride (Mi Paste Plus™) and a varnish containing β-TCP-F and 22,600 ppm sodium fluoride (Clinpro™ White Varnish), and their effects on human enamel were assessed over 5, 10, and 15 days.

Amorphous calcium phosphate (ACP) serves as a fundamental building block for mineralization in both bone and teeth. As a precursor to hydroxyapatite, ACP solutions are rich in calcium and phosphate ions, existing as highly hydrated clusters capable of delivering these ions to repair damaged enamel. The addition of casein phosphopeptide (CPP) further enhances the bioavailability of these ions. CPP, with its four to seven phosphate groups, can bind to ACP nanoclusters, effectively stabilizing and delivering them to the tooth surface. Crucially, the incorporation of fluoride further promotes the uptake of these ions by the enamel [9].

In contrast, β-tricalcium phosphate (β-TCP-F) in the Clinpro™ White Varnish employs a different mechanism. Upon application, saliva breaks down the varnish matrix, providing the tooth with immediate access to calcium ions, phosphate groups, and a high concentration of sodium fluoride (22,600 ppm, equivalent to 50 mg). This varnish utilizes an alcohol-based solution of modified resins and β-TCP-F, and notably includes xylitol. Xylitol is recognized for its significant preventive properties against biofilm formation, primarily by inhibiting the accumulation of *Streptococcus mutans* and *Lactobacillus acidophilus*. While indicated for dentin hypersensitivity, its efficacy in treating initial caries lesions is also under investigation. Some research suggests that high concentrations of sorbitol and xylitol can form Ca^2+^ polyol complexes in saturated calcium sulfate solutions, potentially influencing calcium bioavailability in saliva and promoting remineralization in deeper enamel layers. However, the manufacturer of the Clinpro™ White Varnish does not disclose the specific concentration of xylitol [32]. Given the distinct mechanisms of action of CPP-ACP-F and β-TCP-F, this in vitro study compared their remineralizing potential on artificially induced enamel lesions using the cyclical pH model.

To comprehensively simulate the dynamic oral environment within our in vitro cyclical pH model, the concentration of fluoride ions in both the demineralizing and remineralizing solutions was meticulously measured daily throughout the 15-day treatment period. This approach provides a valuable insight into the potential systemic fluoride exposure that might occur due to the swallowing of saliva containing residual treatment agents. To our knowledge, no prior studies have reported such detailed longitudinal fluoride measurements in this type of experimental setup. Our analysis revealed a significantly higher average fluoride ion concentration (40 ppm) in the demineralizing solutions used for the varnish treatment (β-TCP-F) compared to the paste treatment (CPP-ACP-F). This finding directly correlates with the higher sodium fluoride concentration in the varnish (22,600 ppm) compared to the paste (900 ppm). While the high fluoride concentration in the varnish may contribute to its remineralizing efficacy in vitro, it also raises a crucial clinical concern. The potential for increased systemic fluoride absorption with varnish application warrants careful consideration, particularly in children aged 3 to 8 years during permanent dentition development, where excessive fluoride exposure can lead to dental fluorosis [21].

Such findings were not reported in other in vitro research using a 96 h demineralization model that reported comparable efficacy between β-TCP-F and CPP-ACP-F. However, it is important to mention that different concentration ranges were employed in that study (30–90 mM) [33]. Considering these variations in experimental parameters, future research stemming from our work could explore the impact of varying the concentrations of both treatments to determine if significant changes in the fluoride ion concentration and remineralization efficacy occur.

Currently, the effectiveness of remineralizing agents in treating early caries lesions in vitro [18,26] and managing erosive wear [26] is well documented. While the literature supports the efficacy of CPP-ACP-F in dental remineralization, often enhanced by the addition of fluoride, our present study uniquely contributes to this understanding by specifically investigating the surface modifications induced on lesioned human enamel following treatment. To comprehensively evaluate the effectiveness of the remineralization treatments, we assessed both roughness and hardness parameters, as these are critical indicators of healthy enamel integrity and function [34]. Specifically, Vickers hardness testing was employed as an experimental method to determine the resistance of the enamel tissue to scratching or penetration under a controlled 10 N load across all experimental groups. Our initial measurements confirmed that the hardness values of healthy enamel were significantly higher than those of demineralized (lesioned) enamel. This reduction in hardness is likely a consequence of mineral ion loss during demineralization, leading to weakened surface bonds. Following the application of CPP-ACP-F and β-TCP-F to the demineralized human dental enamel and subsequent exposure to mineralizing–demineralizing pH cycling conditions for 5, 10, and 15 days (mimicking the oral environment), the enamel exhibited a partial recovery of its hardness. The CPP-ACP-F-treated samples showed a slightly greater increase in hardness compared to those treated with β-TCP-F, suggesting a potentially more effective remineralization of the enamel’s mechanical properties.

Our findings, demonstrating a partial recovery of enamel hardness with both treatments and a slightly greater increase with CPP-ACP-F compared to β-TCP-F, align with the results reported by Bhat et al. [34], who also found CPP-ACP-F to be superior using the same treatment agents. While there were differences in the experimental design—Bhat et al. employed a longer 72 h demineralization process and a 28-day treatment duration—the most notable methodological distinction lies in the hardness assessment. They evaluated microhardness, while our study focused on macrohardness using a 10 N load (equivalent to 1.019 kg). Both microhardness and macrohardness, however, serve as indicators of the material’s resistance to indentation. Therefore, the consistency in the observed superiority of CPP-ACP-F across these two studies, despite variations in experimental parameters and hardness assessment techniques, provides a more robust and comprehensive understanding of the effects of these remineralizing agents on enamel.

Regarding the surface roughness of the samples, healthy dental enamel exhibited a lower roughness (Ra = 0.88 μm) compared to demineralized enamel (Ra = 1.33 μm). This increase in roughness in the demineralized group confirms the surface erosion and development of an incipient lesion resulting from exposure to the acidic environment (pH = 4.4) of the demineralizing solution [4]. Following a 15-day treatment with CPP-ACP-F, the surface roughness of the lesioned enamel significantly decreased to Ra = 0.76 μm, effectively reaching levels comparable to those of healthy enamel. This substantial reduction strongly suggests that CPP-ACP-F was successful in remineralizing the damaged enamel surface. In contrast, the treatment with β-TCP-F resulted in only a modest reduction in the roughness of the lesioned enamel (Ra = 1.193 μm) after 15 days, potentially indicating a comparatively lower degree of remineralization achieved by β-TCP-F [22]. This reduction in roughness observed with both treatments can be attributed to the adsorption and deposition of ions onto the irregular surface morphology of the lesioned enamel, effectively filling the demineralization defects.

Wettability, a significant surface property influenced by surface energy and roughness, was evaluated in this study using deionized water to characterize the hydrophobic/hydrophilic nature of the enamel surfaces. Based on the classic surface classification (water contact angle (WCA) > 90° considered hydrophobic), all experimental surfaces exhibited a hydrophilic character. This finding supports the idea that the application of both studied products may enhance the enamel’s resistance to acidic attacks. However, applying Vogler’s definition [35], which sets a biological context limit of WCA 65° to distinguish between hydrophilic and hydrophobic surfaces, reveals a more nuanced picture. The enamel surfaces treated with CPP-ACP-F paste are the only ones showing WCAs slightly below 65°, thus classifying them as hydrophilic under this biological context. This hydrophilic character could potentially correlate with the observed results regarding biofilm formation. The wettability of a surface is determined by the interplay of its microroughness, chemical composition, and intrinsic surface energy, and these properties are also known to influence bacterial adhesion [36]. Generally, increased microroughness and wettability tend to promote bacterial adhesion.

As expected, *Streptococcus mutans* biofilm formation was significantly higher on lesioned enamel surfaces compared to healthy enamel. Notably, a sustained protective effect against biofilm formation was observed following 15 days of treatment of the lesioned enamel with β-TCP-F, resulting in biofilm levels comparable to those on healthy enamel. This sustained reduction in biofilm is likely attributable to the similar surface properties, particularly wettability, achieved between the β-TCP-F-treated and healthy enamel. In contrast, while lesioned enamel surfaces treated with CPP-ACP-F initially showed a comparable anti-biofilm effect to healthy enamel after 5 days, this protection was not sustained at 10 and 15 days. The higher hydrophilic wettability of these CPP-ACP-F-treated surfaces, as previously discussed, may have facilitated the initial adhesion and subsequent spread of *S. mutans*, leading to enhanced colonization over time. Nonetheless, it is important to note that bacterial adhesion is a complex process that is also dependent on bacterial characteristics, such as size and cell envelope structure, and environmental factors, like pH, temperature, ions, and biomolecules.

Consistent with existing research [16,37], our findings indicate that both the CPP-ACP-F paste and β-TCP-F varnish treatments contribute to making acidic attacks from the oral environment less aggressive and enhance the remineralization of surface enamel, thereby preventing initial caries lesions. Supporting this, Tibba et al. [38] evaluated the effect of Silver Diamine Fluoride (SDF) varnish (Riva Star) and sodium fluoride varnish on biofilm formation and other properties. Their results demonstrated a significant improvement in hardness with Riva Star application and a 25–35% reduction in oral biofilm formation with both Riva Star and NaF varnish. In our present investigation, we observed that β-TCP-F treatment for 10 and 15 days, and CPP-ACP-F treatment for 5 days, effectively protected the lesioned enamel surfaces against the initial biofilm growth of *S. mutans*, achieving a similar level of protection as that observed on healthy dental enamel. This highlights the potential of both treatment modalities in mitigating early biofilm development, although the specific mechanisms and long-term efficacy may differ from those observed with SDF and NaF varnishes.

As our hardness results indicate, applying a fluorine-based varnish or paste to lesioned dental enamel is indeed very useful for the remineralization process, considerably increasing the enamel’s hardness values. This highlights the benefit of using a fluorinated and remineralizing compound compared to no treatment. The mechanism of CPP-ACP-F on the enamel surface is often compared to the remineralizing effect of saliva, as both deliver calcium and phosphate ions to the tooth surface, promoting mineral deposition. However, accurately replicating the complex dynamics of in vivo biofilm formation and its potential to directly influence ionic changes in the oral environment within an in vitro setting presents significant challenges. Therefore, the absence of a mature biofilm and the potentially different availability or dynamics of calcium and phosphate ions compared to the constant, low-level supersaturation provided by saliva in vivo might have influenced the extent to which the CPP-ACP-F system could exert its full remineralizing potential in our in vitro model [39].

While the remineralizing effect of CPP-ACP-F on the enamel surface shares similarities with that of saliva, the complex in vivo environment, characterized by the presence of a biofilm that directly influences ionic exchanges, is challenging to fully replicate in vitro. The absence of this biofilm and the artificially optimized conditions in our experimental setup, potentially leading to a state of supersaturation of calcium and phosphate ions, might have influenced the remineralizing action of the CPP-ACP-F system compared to its performance under natural oral conditions. Recognizing these inherent limitations of our in vitro model, which does not account for factors such as variable saliva composition, dietary intake, hygiene habits, medication use, and the dynamic nature of the oral microbiota, our findings represent an initial yet promising step towards assessing the remineralizing effects of β-TCP-F and CPP-ACP-F. To gain a more realistic understanding of how these two products impact tooth enamel health, future research should focus on conducting evaluations in in vivo models and clinical studies that can better capture the complexities of the oral environment.

## 5. Conclusions

In conclusion, both the β-TCP-F varnish and CPP-ACP-F paste treatments demonstrated the ability to counteract surface modifications on human enamel caused by in vitro demineralization. Both compounds increased the hardness of lesioned human enamel to levels comparable to healthy enamel. However, regarding surface roughness, only the lesioned enamel treated with the CPP-ACP-F paste achieved roughness levels similar to healthy enamel. Microbiological in vitro studies revealed that the demineralized enamel treated with β-TCP-F varnish provided sustained anti-biofilm protection comparable to healthy enamel throughout the study period, whereas the CPP-ACP-F paste’s anti-biofilm effect was only significant at the beginning of the treatment. Importantly, our findings regarding fluoride concentration in the pH cycle solutions suggest that caution may be warranted when using β-TCP-F varnish due to the high amount of fluoride ions released into the aqueous media, potentially limiting its frequent application in very young patients to mitigate the risk of dental fluorosis.

Overall, the application of both studied remineralizing products on lesioned human dental enamel appears beneficial in preventing the damages produced by demineralization and may be recommended for patients with a high risk of caries and for preventive purposes. Considering the global public health concern of dental caries, characterized by enamel damage, the study of remineralizing agents is crucial to support their evidence-based use in dental practice and by consumers. In this context, the findings reported in this work contribute to the growing body of evidence supporting the efficacy of these products in managing early caries lesions.

## Figures and Tables

**Table 1:** Vickers hardness number (mean ± SD) of human enamel specimens of each group studied.

Groups	Vickers Hardness Number (HV)			
	0 Days	5 Days	10 Days	15 Days
Group I: HE	533.8 ± 14.8 HV			
Group II: IL	281.0 ± 04.1 HV			
Group III: β-TCP-F		448.2 ± 09.8 HV	406.3 ± 22.1 HV	473.6 ± 12.8 HV
Group IV: CPP-ACP-F		448.1 ± 10.9 HV	464.6 ± 11.6 HV	515.2 ± 10.7 HV

Group I: HE (healthy enamel), Group II: IL (initial lesion), Group III: β- TCP-F (β-tricalcium phosphate with NaF), and Group IV: CPP-ACP-F (casein phosphopeptide-amorphous calcium phosphate with NaF).

**Table 2:** Roughness (Ra) results (mean ±SD) of the human enamel specimens of different groups.

Groups	Ra (μm) 0 Days	Value *p*	Ra (μm) 5 Days	Value *p*	Ra (μm) 10 Days	Value *p*	Ra (μm) 15 Days	Value *p*
Group I: HE	0.88 (±0.08)	0.74						
Group II: IL	1.33 (±0.14)	0.62						
Group III: β-TCP-F			1.358 (±0.25)	0.96	1.29 (±0.13)	0.48	1.193 (±0.14)	0.30
Group IV: CPP- ACP-F			0.72 (±0.05)	0.13	0.68 (±0.01)	0.90	0.76 (±0.05)	0.00

Ra: Roughness media. Group I: HE (healthy enamel), Group II: IL (initial lesion), Group III: β-TCP-F treatment, and Group IV: CPP-ACP-F treatment. One-way analysis of variance (ANOVA).

**Table 3:** Water contact angles (°) results (mean ± SD) of the human enamel specimens of different groups.

Groups	Water Contact Angle (°)			
	0 Days	5 Days	10 Days	15 Days
Group I: HE	73.65 ± 0.72			
Group II: IL	80.82 ± 0.15			
Group III: β-TCP-F		78.26 ± 0.72	73.54 ± 0.40	71.01 ± 0.69
Group IV: CPP-ACP-F		60.42 ± 0.18	63.95 ± 0.21	65.24 ± 1.57

Group I: HE (healthy enamel), Group II: IL (initial lesion), Group III: β-TCP-F (β-tricalcium phosphate with NaF), and Group IV: CPP-ACP-F (casein phosphopeptide-amorphous calcium phosphate with NaF).

**Table 4:** Physicochemical characterization results of the two experimental groups in each test are compared to highlight the relative effectiveness of each treatment.

	ppm F *	HV ^&^	Ra (μM) ^+^	WCA (°) ^#^
β-TCP-F 5 DAYS	--	448.2 ± 09.8	1.358 ± 0.25	78.26 ± 0.72
β-TCP-F 10 DAYS	--	406.3 ± 22.1	1.29 ± 0.13	73.54 ± 0.40
β-TCP-F 15 DAYS	--	473.6 ± 12.8	1.193 ± 0.14	71.01 ± 0.69
CPP-ACP-F 5 DAYS	--	448.1 ± 10.9	0.72 ± 0.05	60.42 ± 0.18
CPP-ACP-F 10 DAYS	--	464.6 ± 11.6	0.68 ± 0.01	63.95 ± 0.21
CPP-ACP-F 15 DAYS	72 sol rem	515.2 ± 10.7	0.76 ± 0.05	65.24 ± 1.57

*: fluoride release, NaF, ppm, cumulative fluoride ions released over 15 days; &: Vickers hardness, HV; +: roughness, Ra, (μm); #: water contact angle (WCA °).

## Data Availability

The data presented in this study are available upon request from the corresponding author.
